# Antibody-drug conjugates in breast cancer: overcoming resistance and boosting immune response

**DOI:** 10.1172/JCI172156

**Published:** 2023-09-15

**Authors:** Hannah L. Chang, Blake Schwettmann, Heather L. McArthur, Isaac S. Chan

**Affiliations:** 1 Department of Internal Medicine, Division of Hematology and Oncology,; 2 Harold C. Simmons Comprehensive Cancer Center, and; 3 Department of Molecular Biology, University of Texas Southwestern Medical Center, Dallas, Texas, USA.

## Abstract

Antibody-drug conjugates (ADCs) have emerged as a revolutionary therapeutic class, combining the precise targeting ability of monoclonal antibodies with the potent cytotoxic effects of chemotherapeutics. Notably, ADCs have rapidly advanced in the field of breast cancer treatment. This innovative approach holds promise for strengthening the immune system through antibody-mediated cellular toxicity, tumor-specific immunity, and adaptive immune responses. However, the development of upfront and acquired resistance poses substantial challenges in maximizing the effectiveness of these therapeutics, necessitating a deeper understanding of the underlying mechanisms. These mechanisms of resistance include antigen loss, derangements in ADC internalization and recycling, drug clearance, and alterations in signaling pathways and the payload target. To overcome resistance, ongoing research and development efforts are focused on urgently identifying biomarkers, integrating immune therapy approaches, and designing novel cytotoxic payloads. This Review provides an overview of the mechanisms and clinical effectiveness of ADCs, and explores their unique immune-boosting function, while also highlighting the complex resistance mechanisms and safety challenges that must be addressed. A continued focus on how ADCs impact the tumor microenvironment will help to identify new payloads that can improve patient outcomes.

## Introduction

Antibody-drug conjugates (ADCs) are a class of therapeutics that have been developed to increase the specificity of cytotoxic therapy by linking cytotoxic molecules to a targeted protein carrier. A linker connects the cytotoxic drug to the protein carrier and can be non-cleavable or designed with specific release mechanisms to allow for controlled cleavage inside the tumor cell, rendering the linker cleavable (1). Through this unique drug design, ADCs deliver highly potent cytotoxic drugs, or payloads, in a targeted fashion to antigen-expressing cells. In breast cancer, this class of biopharmaceutical drugs has revolutionized treatment because it enables targeted therapy across multiple breast cancer subtypes (2, 3). Traditionally, breast cancer therapies have relied on categorizing the disease into subtypes, determined by the presence of certain proteins, such as estrogen receptor, progesterone receptor, and human epidermal growth factor receptor 2 (HER2). Triple-negative breast cancer (TNBC) is another subtype, defined by the lack of expression of these antigen targets.

## Current landscape

The first ADCs to reach FDA approval were developed for the treatment of hematologic malignancies. In 2013, the first approved ADC for solid tumors, an anti-HER2 ADC, trastuzumab emtansine (T-DM1), gained FDA approval for patients with metastatic HER2-positive breast cancer. This approval was based on the EMILIA trial, a phase III trial comparing T-DM1 to lapatinib plus capecitabine in patients with metastatic HER2-positive breast cancer who were previously treated with trastuzumab and a taxane-based therapy (4, 5). Since then, T-DM1 has been brought into the adjuvant setting, and two additional ADCs, trastuzumab deruxtecan-nxki (T-DXd) and sacituzumab govitecan-hziy (SG), have been approved for the treatment of breast cancer in the metastatic setting (3, 6–8). While also directed against HER2, T-DXd conjugates trastuzumab to a topoisomerase I (TOP1) inhibitor, exatecan payload, through a stable protease-sensitive cleavable linker (9). The improved efficacy can likely be explained, in part, by a higher drug-to-antibody ratio coupled with T-DXd’s tumor-selective cleavable linker, which is an enzymatically cleavable peptide that is decomposed by intratumoral lysosomes (10, 11). SG consists of a trophoblast cell surface antigen 2 (TROP2) antibody that is linked to SN-38, the active metabolite of irinotecan, via a hydrolyzable linker. Several ADCs have undergone trials for the treatment of breast malignancies that have led to their approval (Table 1). These trials have also been reviewed extensively elsewhere (12–14).

## Mechanisms of action and key elements

Manufacture of ADCs is typically completed through three processes: generation of the antibody, synthesis of the small-molecule drug, and conjugation of those two components (15). An ADC directs its payload to cancer cells through binding of the monoclonal protein carrier to cell surface target antigens. The ADC receptor complex is subsequently internalized via antigen-mediated endocytosis and undergoes lysosomal processing during which the payload is released into the intracellular space. The expression levels of a target antigen can vary greatly within each tumor and between different lesions within the same individual. Certain payloads possess membrane permeability and can diffuse to neighboring cells, irrespective of the expression of the target antigen, resulting in a cytotoxic effect. This phenomenon, known as the “bystander effect,” provides an additional advantage by addressing tumor antigen heterogeneity (16).

Through a process called conjugation, the antibody is linked to a synthetic molecule, which is typically a small-molecule drug (15). During conjugation, the drug-to-antibody molar ratio (DAR) is determined through liquid chromatography–mass spectrometry. The DAR is critical to ensure that enough drug is appended to specific regions of the antibody (17). Because the linkage of the drug to the antibody is stochastic in nature, there can be drastic changes in efficacy based on whether the drug is insufficient, overabundant, or on nonspecific sites on the antibody. The design and mechanisms of actions of ADCs have been exhaustively reviewed in previously published reviews on ADCs (18–21).

## Toxicity

When normal tissues express the target antigen, target-mediated endocytosis can lead to direct toxicity to normal tissues (22). However, the majority of dose-limiting toxicity is related to target-independent uptake mechanisms into normal cells (22). The mechanisms underlying these observations encompass ADC uptake through the Fcγ receptor (FcγR) on normal cells, nonspecific endocytic processes in normal cells, and linker-drug stability (22). In the investigation of T-DXd lung toxicity using monkey models, diffuse lung toxicity affecting the alveolar space was observed, rather than being localized to HER2-expressing bronchial cells (23). This observation suggests that lung toxicity associated with T-DXd may not be dependent on HER2-mediated uptake. Instead, target-independent uptake by normal cells and the stability of the payload linker likely have a more important impact. T-DXd primarily accumulates in alveolar macrophages in monkey lungs (23), and the protease cathepsin B, expressed in these macrophages, is known to be responsible for cleaving the linker of T-DXd (11). Hence, the uptake of T-DXd by macrophages and subsequent cathepsin-mediated payload cleavage may account for the increased rates of T-DXd lung toxicity.

## Mechanisms of resistance and strategies to overcome resistance

**Figure 1:**
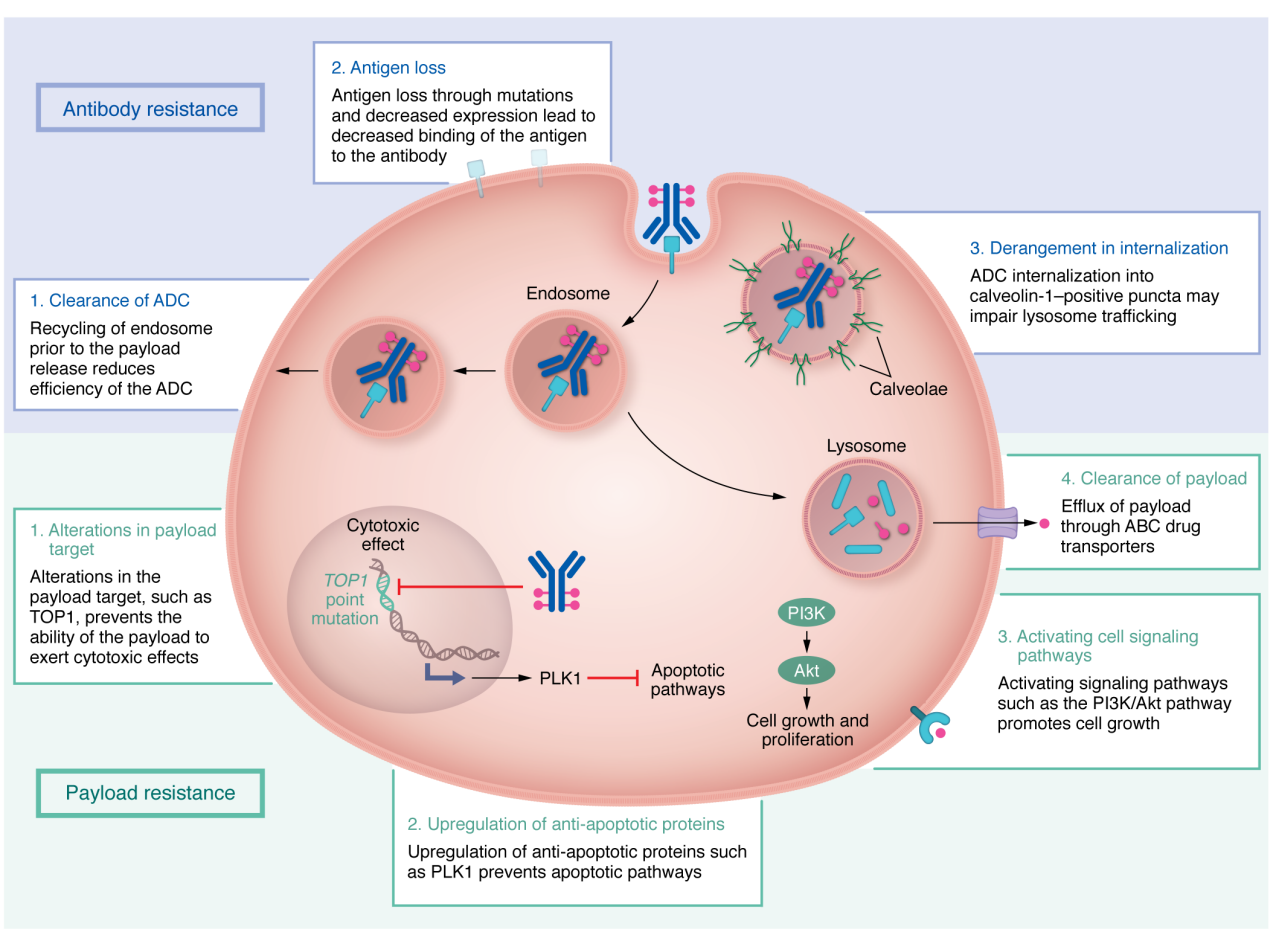
Mechanisms of resistance. Resistance mechanisms to antibody-drug conjugates (ADCs) can be broadly classified into two categories: antibody resistance and payload resistance. Antibody resistance mechanisms include clearance of ADC, antigen loss, and derangement in internalization. Payload resistance mechanisms include alterations in the payload target, upregulation of antiapoptotic proteins, activation of cell signaling pathways, and clearance of the payload.

Despite the clinical impact of ADCs, a portion of patients have de novo or primary resistance to ADCs, and another subset of patients initially respond to ADCs but later develop resistance. Several trials have reported disease progression without an initial tumor response to ADCs in breast cancer (Table 2). Mechanisms of ADC resistance are complex but are generally related to resistance against the antibody or payload component (Figure 1). Resistance to the entire ADC complex can occur through physical barriers, such as the binding site barrier (24). This barrier is the result of a dense tumor microenvironment that prevents the ADC from distributing throughout the tumor at the dose used for ADCs. Physical barriers can also be seen with ADCs targeting brain tumors due to their trouble passing the blood-brain barrier (25). Other factors like absorption, distribution, metabolism, and excretion (collectively known as ADME) behaviors, DAR value, and dosage can also contribute to resistance. ADME behaviors can result in lower amounts of circulating ADC, like in the case of an h1F6-targeting antibody that is cleared faster when conjugated to a chemotherapeutic payload (26). Higher DAR values can also increase clearance from the body (26). Dose-related mechanisms of resistance have been further investigated in dose escalation studies or via a cyclical method in which treatment of tumors occurs at high doses for short periods (27).

### Antibody resistance

#### Antigen loss.

Several studies have shown that with exposure to ADCs, there is a marked decrease in antigen levels shortly following initiation of treatment (28). Decreased HER2 was seen in human breast cancer cell lines at the protein and RNA levels upon chronic exposure to T-DM1, suggesting a transcriptional mechanism of HER2 downregulation (28). Although treatment with ADCs could select for clones of antigen-negative cancer cells, the bystander effect of ADCs could potentially alleviate clonal expansion of resistant cells. However, artificial CRISPR/Cas9–mediated *TROP2* deletion in TNBC cells has been shown to suppress TNBC cell growth (29). Downregulation of TROP2 through the generation of small hairpin RNA targeting *TROP2* also decreased the invasion ability of the TNBC cell line, suggesting that antigen downregulation in response to TROP2 ADC treatment could potentially impair cancer cells (29). However, the impact of TROP2 loss under anti-TROP2 pressure has not been explored; thus, the translational benefit of these findings remains unclear.

In addition to ADC-targeted destruction of antigen-expressing cells, antigen loss has also been shown to result from acquired molecular alterations in the antibody target. Studies specifically observed acquired molecular alterations in the antibody target TROP2 in patients who experienced a prolonged response but eventually progressed on SG. The TACSTD2/TROP2T256R missense mutation encodes a protein that has markedly lower binding affinity to the antibody, which can explain one mechanism of resistance (30). Truncated forms of the antigen are another potential mechanism of resistance. While resistance to trastuzumab has been associated with a truncated form of HER2, p96HER2, it is not yet clear whether p96HER2 also reduces binding to anti-HER2 ADCs like T-DM1 and T-DXd (31).

#### Derangement of ADC internalization and recycling.

Cancer cells can also develop resistance through derangements in ADC internalization and trafficking to lysosomes. Endocytosis followed by lysosomal degradation is the central route by which ADCs are processed. Investigators evaluating T-DM1 resistance found that in resistant cells, ADCs are instead internalized into caveolin-1–positive (CAV-1–positive) puncta with altered trafficking to lysosomes. Internalization into CAV-1–positive puncta does not allow for appropriate enzymatic processing of the non-cleavable linker of T-DM1. Additionally, because these CAV-1–positive compartments have a neutral pH, charged payloads are unable to permeate through the membrane to act on neighboring cells, thus reducing bystander activity of the ADC (32).

Even when ADCs are appropriately internalized, a percentage of these endosomes are rapidly recycled back to the cell membrane before release of the payload, leading to clearance of the ADC out of the cell (33). Increased lysosomal pH was found to prevent the proteinase activity of lysosomal enzymes and subsequently decrease the activity of T-DM1 (34).

One strategy to overcome these mechanisms of resistance is to promote more rapid internalization and effective lysosomal trafficking. Bivalent biparatopic HER2-directed ADCs are designed to address this hypothesis by targeting two non-overlapping epitopes on HER2 to induce HER2 clustering (35). Theoretically, this design creates a large meshwork of receptor clustering and results in rapid and enhanced HER2 internalization versus any monospecific antibody. Biparatopic antibodies also lead to increased lysosomal degradation (36, 37). This approach is being tested in phase I clinical studies (38).

### Payload resistance

#### Drug clearance.

Drug clearance through increased expression of drug efflux pumps is one of the most studied pathways of chemotherapy resistance (39). Similar mechanisms have been evaluated with ADCs given that the primary mechanism of action is through the delivery of chemotoxic payloads. In cell lines resistant to brentuximab vedotin, the ATP-binding cassette (ABC) drug transporter ABCB1 has been shown to be upregulated, increasing the export of the payload monomethyl auristatin E (MMAE) out of the cell (40). Similarly, studies looking at mechanisms of resistance to the anti–nectin-4 ADC enfortumab vedotin-ejfv also found upregulation of *ABCB1* expression in resistant tumors (41). Sensitivity to enfortumab vedotin-ejfv has been shown to be restored through newer-generation ABC transport inhibitors, such as tariquidar, which targets ABCB1 (41). Additionally, clearance by efflux pumps is affected by the type of payload used. For example, ABCB1-overexpressing cells exposed to an MMAE payload had dramatically reduced MMAE activity (42). In contrast, the activity of the payload monomethyl auristatin F (MMAF) was not affected by the ABCB1-overexpressing cell line (42). The difference in susceptibility of these compounds to efflux may be related to alkyl substitutions of large size on MMAF (42).

#### Alterations in signaling pathways.

As with many other cancer chemotherapeutics, ongoing exposure to ADCs can lead to selective pressure for acquired resistance mutations. Studies evaluating gemtuzumab ozogamicin resistance in acute myeloid leukemia (AML) cells have shown activation of the phosphatidylinositol-3-kinase (PI3K)/protein kinase B (AKT) pathway, and inhibition of this pathway with the investigational AKT inhibitor MK-2206 was able to sensitize resistant AML cells to calicheamicin-γ, a DNA-binding cytotoxic antibody and the payload of gemtuzumab ozogamicin (43). In breast cancer cells, loss of the tumor suppressor PTEN results in activation of PI3K/AKT signaling. T-DM1–resistant cells were found to have reduced PTEN levels, which supports the finding that PI3K/AKT activation leads to resistance. Combination with a PI3K inhibitor, CDC-0941, led to synergistic inhibition of breast cancer cell growth, suggesting that combination therapy with this class of inhibitors could circumvent ADC resistance (44).

Modulation of the apoptotic signaling pathway is another pathway of resistance. Polo-like kinase 1 (PLK1), a mitotic kinase that regulates the cell cycle, is upregulated in T-DM1–resistant cells (45). Inhibition of PLK1 with volasertib reversed resistance by inducing spindle assembly checkpoint–dependent mitotic arrest, followed by cyclin-dependent kinase-1 (CDK1) phosphorylation and inactivation of the antiapoptotic protein B cell lymphoma 2 (Bcl-2) (45). In T-DM1–resistant cells, overexpression of the leukemia inhibitory factor receptor (LIFR) has been shown to activate the signal transducer and activator of transcription 3 (STAT3) pathway (46). This cascade led to upregulation of the antiapoptotic proteins Bcl-xL, Bcl-2, survivin, and Mcl-1, and subsequently conferred T-DM1 resistance (46). STAT3 activation was overcome by napabucasin, a STAT inhibitor, suggesting that this pathway is another potential target (46).

Targeting the DNA damage response pathway in combination with ADCs is another approach to overcome payload resistance. As a part of the DNA damage response pathway, DNA-topoisomerase complexes are usually removed by poly(ADP-ribose) polymerase–dependent (PARP-dependent) mechanisms, which allows the DNA breaks to be repaired. PARP inhibitors block this pathway to allow for persistent DNA strand breaks and subsequent cell death. Phase I/II trials combining SG and the PARP inhibitors talazoparib and rucaparib have shown promising synergistic antitumor effects (47). Tolerability may be an issue with this combination, and further investigation may help in determining the optimal dose and schedule.

#### Alterations in payload target.

Studies have also found alterations in the payload target TOP1 following exposure to T-DM1. The point mutation of *TOP1* is believed to alter the DNA binding affinity of the enzyme and prevent adequate binding of the payload to the enzyme-DNA interface (30). These findings are not unique to ADCs and are consistent with historical reports of alterations in TOP1 that confer resistance to conventional chemotherapy treatments using TOP1 inhibitors (48).

Payload diversification may be one way to overcome payload resistance due to alterations in the payload target. NCT04152499 (ClinicalTrials.gov) is a phase I/II clinical trial evaluating a TROP2-targeted ADC, with a belotecan derivative payload. Patients with HR^+^/HER2^–^ advanced or metastatic breast cancer who previously received therapy with an anti-HER2 ADC (T-DXd) for HER2-low tumors are eligible. The sequential administration of ADCs with distinct mechanisms of action for their payloads holds the potential to overcome resistance to payload alterations.

#### Urgent need for biomarkers.

Given that response rates to ADCs can be as low as 21% in trials, there is a need for improved ADC biomarkers that appropriately select patients and improve the therapeutic index of these drugs. The FDA recently approved a companion diagnostic based on immunohistochemistry (IHC) to aid the assessment of HER2-low breast cancer and to predict response to T-DXd in this population (49). However, there are no approved biomarkers that can predict which patients will develop resistance to ADCs. Traditionally, biomarkers are clinically detected by IHC expression or other assays such as FISH, although both methods have their own limitations, including sampling error in focally receptor-positive tumors and limited reproducibility of receptor assays (50, 51). Newer methods can involve imaging and circulating tumor products (52).

However, biomarker development for ADCs must overcome several challenges. Upon evaluation of phase I and II clinical trials conducted with ADCs using preselected target antigens, 19% of the trials showed no relationship between target expression and response to the ADC (53). This finding suggests that the presence of target expression is only one important component of patient selection. Instead, payload resistance and response markers, such as tubulin-β3 (TUBB3) and TOPO1, are being evaluated on formalin-fixed, paraffin-embedded tissue from pancreatic adenocarcinoma tumors and colorectal cancer tumors (54, 55).

Other challenges to ADC biomarker development include tumor heterogeneity and the accuracy of sampling from one biopsy at one moment in time (56). Newer modes of assessing protein include imaging of radiolabeled monoclonal antibody (mAb) by positron emission tomography (PET), termed “immuno-PET.” These methods quantify the mAb in tissues and could be used to improve the therapeutic window of ADCs through dose optimization (57). In the ZEPHIR trial for breast cancer, baseline Zr-trastuzumab imaging was obtained before T-DM1 therapy followed by computerized tomography (CT) scans to assess response (58). Of patients classified as HER2 positive by immuno-PET, 72% had a positive response in imaging. Conversely, of patients classified as HER2 negative by immuno-PET, 88% had stable or progressive disease (58).

Detection and measurement of antigens on circulating tumor cells (CTCs) is another noninvasive companion diagnostic tool to identify patients likely to benefit from treatment with ADCs. An immunofluorescence-based assay has been developed to evaluate the target antigen of six-transmembrane epithelial antigen of prostate 1 (STEAP1) on CTCs in metastatic castration-resistant prostate cancer patients (59). In a phase I study evaluating the ADC targeting STEAP1 in metastatic castration-resistant prostate cancer, there was general consistency between CTCs, the prostate cancer screening antigen PSA, and response on imaging (60).

Because of its ease of collection, circulating tumor DNA (ctDNA) is rapidly being integrated clinically as a noninvasive biomarker for cancer diagnosis, staging, treatment response monitoring, and residual disease (61). The phase II HERALD trial administered T-DXd to patients with an advanced solid tumor malignancy with HER2 amplification as identified by a 74-gene sequencing ctDNA panel, Guardant 360 (62). In this trial, tumor tissue, ctDNA, and CTCs are all being collected to better understand the role of these tests as predictive biomarkers and makers of resistance. While the results have not yet been reported, there is a need for additional studies to inform the role of liquid biopsies in clinical practice.

## Enhancing immune antitumor activity

**Figure 2:**
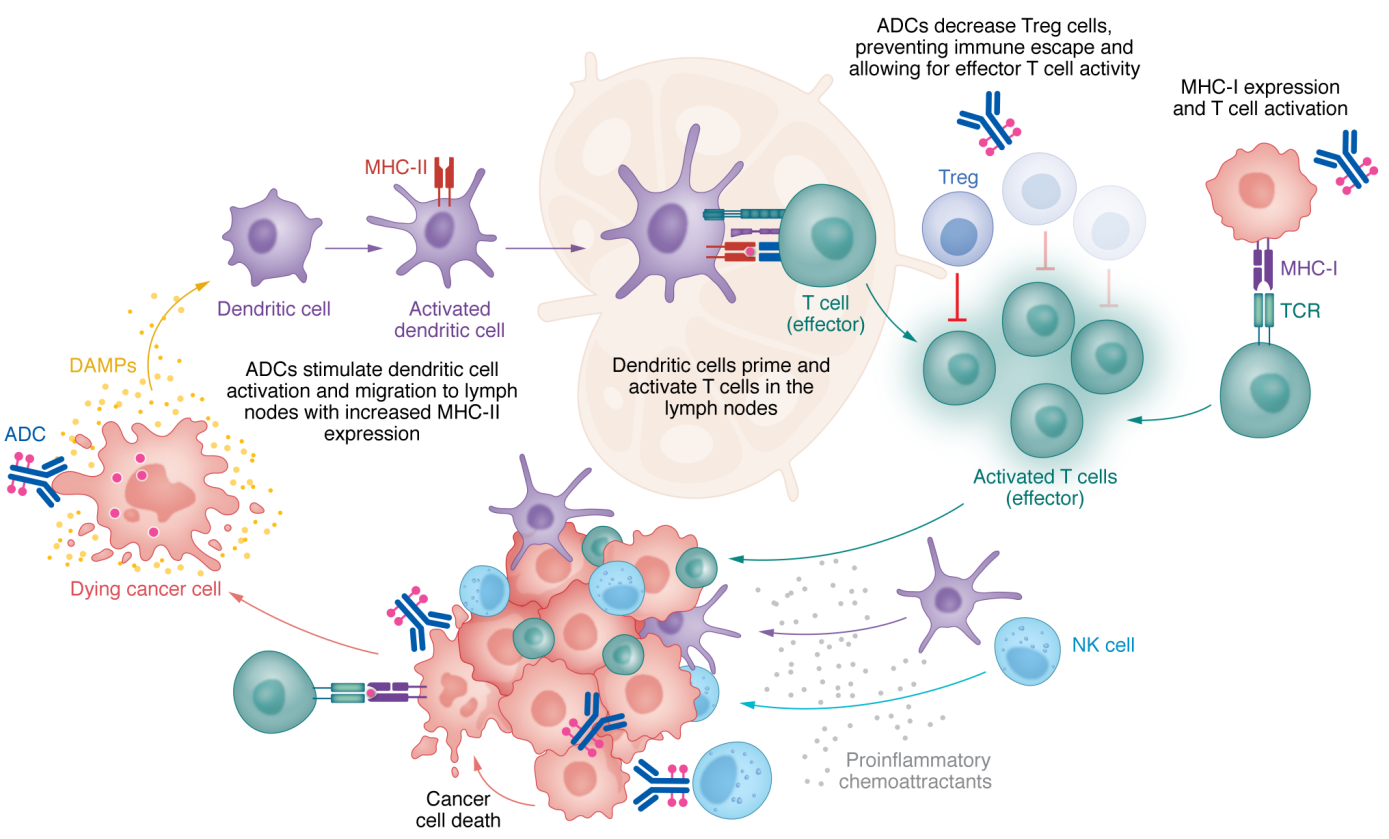
Antibody-drug conjugates in the cancer-immunity cycle. ADCs play a crucial role in activating tumor immunity across all stages of the cancer-immunity cycle. Targeted release of the ADC payload leads to tumor cell death and subsequent release of DAMPs that stimulate activation of dendritic cells. Mature dendritic cells facilitate antigen uptake and migration to lymph nodes. In the lymph nodes, ADCs reduce Tregs and augment MHC-I expression, thereby promoting the effective activity of cytotoxic (effector) T cells. ADCs enhance leukocyte infiltration and expand CD4^+^ and CD8^+^ T cells, NK cells, and IFN-γ production. Secretion of proinflammatory chemoattractants such as IFN-γ recruits immune cells including NK cells, dendritic cells, and CD4^+^ and CD8^+^ T cells. The antibody component of ADCs activates the immune system via antibody-dependent cell-mediated cytotoxicity (ADCC), antibody-dependent cellular phagocytosis (ADCP), and complement-dependent cytotoxicity (CDC).

In addition to cytotoxic benefits of the payload, ADCs engage the immune system across all stages of the cancer-immunity cycle (Figure 2) (63). Like mAbs, ADCs are believed to activate cellular immune defense through antibody-dependent cell-mediated cytotoxicity (ADCC), antibody-dependent cellular phagocytosis (ADCP), and complement-dependent cytotoxicity (CDC). During ADCC, the antibody binds the target antigen through fragment, antigen-binding (Fab). The FcγR portion of effector immune cells binds to the Fc portion of antibodies to orchestrate ADCC that kills antibody-coated target cells through the release of perforin and other cytotoxic granules (64). For example, in NK cells, CD16 is one FcγR that, upon binding, phosphorylates immune tyrosine–based activating motifs (ITAMs), leading to NK cell proliferation and degranulation (65). NK cells are critical immune cells that respond to ADCC initiation by both T-DXd and TDM1, even after payload conjugation (11, 66). In ADCP, the Fc portion of the antibody binds and activates the FcγRs on tumor-associated macrophages (TAMs) to induce phagocytosis and thus degradation of the target cell. Multiple studies have also shown that this mechanism is involved in tumor clearance and the anti-immune effect of mAbs (67, 68). While the contribution of this ADCP to ADC efficacy is not currently known, preclinical studies have shown that ADCs can mediate ADCP (69).

To improve the effectiveness of IgG antibodies, researchers are conducting Fc engineering studies to strengthen the neonatal Fc receptor (FcRn) interactions, which play a crucial role in protecting IgG molecules from degradation and therefore extend the circulating half-life of IgG antibodies (70, 71). However, altering the Fc regions in a way that suppresses FcγR binding can impact the immunogenicity of the modified Fc regions by reducing the binding to C1q, and consequently diminishing effector functions such as ADCC and CDC (71). Therefore, finding a balance between prolonging antibody half-life through FcRn interactions and maintaining optimal binding to other components of the immune system is crucial in the development of these agents.

There is increasing interest in using ADCs to increase tumor-specific immunity. One approach is to use ADCs to target and reduce immunosuppression in the tumor microenvironment (TME). For example, immature dendritic cells (DCs) can be immunosuppressive, and therapeutics that activate their maturation are of interest (72–74). Dolastatins, the payload of brentuximab vedotin, have been shown to induce DC maturation as evidenced by a dose-dependent increase in the DC maturation markers CD80, CD86, CD40, and MHC-II in all tests performed on human cells (75). The payload of brentuximab vedotin has also been shown to promote antigen uptake and migration of DCs to the tumor-draining lymph nodes, although the direct mechanisms underlying this observation remain unclear (75). The payload of T-DXd has also been shown to activate DC maturation through upregulation of CD86 and major MHC-II expression (76). Moreover, ansamitocin P3, the precursor of the payload DM1, facilitates DC activation by stimulating the migration of tumor-resident DCs to tumor-draining nodes (77). In this study, the expression of MHC-II and DC costimulatory molecules, CD80, CD86, and CD40, was significantly increased after ansamitocin P3 exposure in comparison with controls.

In contrast to increased DC maturity to reduce overall immunosuppression in the TME, Tregs were found to be decreased in peripheral blood samples of patients treated with brentuximab vedotin. A shift in the CD3^+^IFN-γ^+^ and CD8^+^IFN-γ^+^ effector T cell (Teff)/CD4^+^CD25^+^FoxP3^+^ Treg ratio further supports the potential role that ADCs play to augment antitumor immune responses by reducing the immunosuppressive contributions from specific regulatory immune cells within the TME (75). There is currently little information on the impact of ADCs on other suppressive immune cells such as myeloid-derived suppressor cells, and future studies will need to be conducted to better understand their interaction.

Additional studies have shown that ADCs augment tumor-specific immunity by altering the pattern of tumor immune cell infiltrates. Leukocyte infiltration is increasingly being recognized as a prognostic and predictive biomarker in breast cancer (78). A study evaluating tumor-infiltrating lymphocytes following administration of T-DM1 found expansion of CD4^+^ and CD8^+^ effector T cells and IFN-γ production. To highlight the importance of T cells for the efficacy of T-DM1, the investigators removed the CD4^+^ and CD8^+^ T cells and revealed that these T-DM1–treated mice had reduced survival time. However, further evaluation of immune cell subsets increased numbers of NK and natural killer T (NKT) cells upon exposure to T-DM1, suggesting that these effector immune cells are also important in the inflammatory response induced by ADCs. Specifically, there was an increase in EOMES-positive NK cells, which exert potent antitumor effector functions (79). T-DM1 was found to increase the NK cell– and T cell–specific proinflammatory chemokines MIG/CXCL9, MIP-1α/CCL3, MIP-1β/CCL4, and RANTES, which could explain the increased NK and T cell numbers (80).

ADCs can also elicit long-lasting antitumor effects through the adaptive immune system via memory T cells and B cells. Immunologic memory plays a crucial role in the control of tumors and maintenance of a therapeutic response. Iwata et al. used syngeneic mouse models inoculated with CT26.WT-hHER2 cells to show that ADCs promote antigen spreading (76). This phenomenon occurs when treatment-induced T cells recognize not only the target antigen but other antigens of the tumor cell. In this study, mice were initially injected with CT26.WT-hHER2 cells and treated with T-DXd once they had a tumor (76). Then treated mice were rechallenged with both HER2-expressing tumors and non-HER2-expressing tumors and rejected both types. This result suggested that T-DXd stimulated immune cells that recognized non-HER2 tumor antigens. Furthermore, in in vitro studies, splenocytes of naive and T-DXd–treated mice were cocultured with both HER2-expressing and non-HER2-expressing tumor cells, and the splenocytes of T-Dxd–treated mice reacted to both tumor cells with increased IFN-γ secretion, whereas the splenocytes of naive mice did not (76).

Given the immunomodulatory effects of ADCs, investigators are evaluating whether the combination of immune checkpoint inhibitors (ICIs) and ADCs can enhance T cell responses and overcome mechanisms of immune resistance. The immune inhibitory receptor cytotoxic T lymphocyte–associated antigen 4 (CTLA-4) was found to be markedly increased on T cells upon T-DM1 treatment. Further, the immune inhibitory ligand PD-L1 was markedly increased on TAMs upon T-DM1 treatment (80). These studies suggest that immune escape can be a potential mechanism of ADC resistance and provide a rationale for combination therapies. Preclinical mouse models have demonstrated that the upfront combination of T-DM1 and dual immune checkpoint blockade (anti–CTLA-4 with anti–PD-1) leads to nearly 100% cure of mice, while either of these therapies alone was much less effective (80). Likewise, T-DXd has also been found to directly increase both MHC class I and PD-L1 expression in mouse models (76). While PD-L1 expression decreases tumor immunity, the increase in MHC class I expression suggests that T-DXd also promotes T cell activation (81). The combination of T-DXd and PD-L1 blockade successfully increased survival of the mouse models compared with monotherapy alone (76). The role of this combination is being further explored in several clinical trials, including NCT03334617, NCT03742102, NCT04379596, NCT03523572, NCT02302339, NCT03288545, NCT02572167, NCT05489211, NCT05039073, NCT04561206, NCT02758717, NCT03057795, NCT01703949, and NCT01896999.

Despite the strong preclinical rationale for ADC and ICI combination therapies, a major concern is the potential for overlapping toxicities. Specifically, T-DXd monotherapy is associated with around 15% incidence of pneumonitis (82), whereas ICI monotherapy shows a lower incidence 3% (83). In the KATE2 trial, the addition of atezolizumab to T-DM1 in TNBC patients resulted in more toxicity, with 33% of patients experiencing serious adverse events. While no clinical benefit was observed in the intention-to-treat population, exploratory analyses suggest a potential benefit in patients with PD-L1–positive TNBC, highlighting the importance of identifying the specific patient subset that may derive benefit from this combination (84). On the other hand, safety data from BEGONIA, which combined Dato-DXd and durvalumab in metastatic TNBC patients, noted no cases of pneumonitis (85). To achieve a balance between effectiveness and tolerability, combination ADC and ICI therapies need to optimize their payloads and dosing strategy, which may require sequential dosing or dose reductions.

## Payloads under investigation

**Figure 3:**
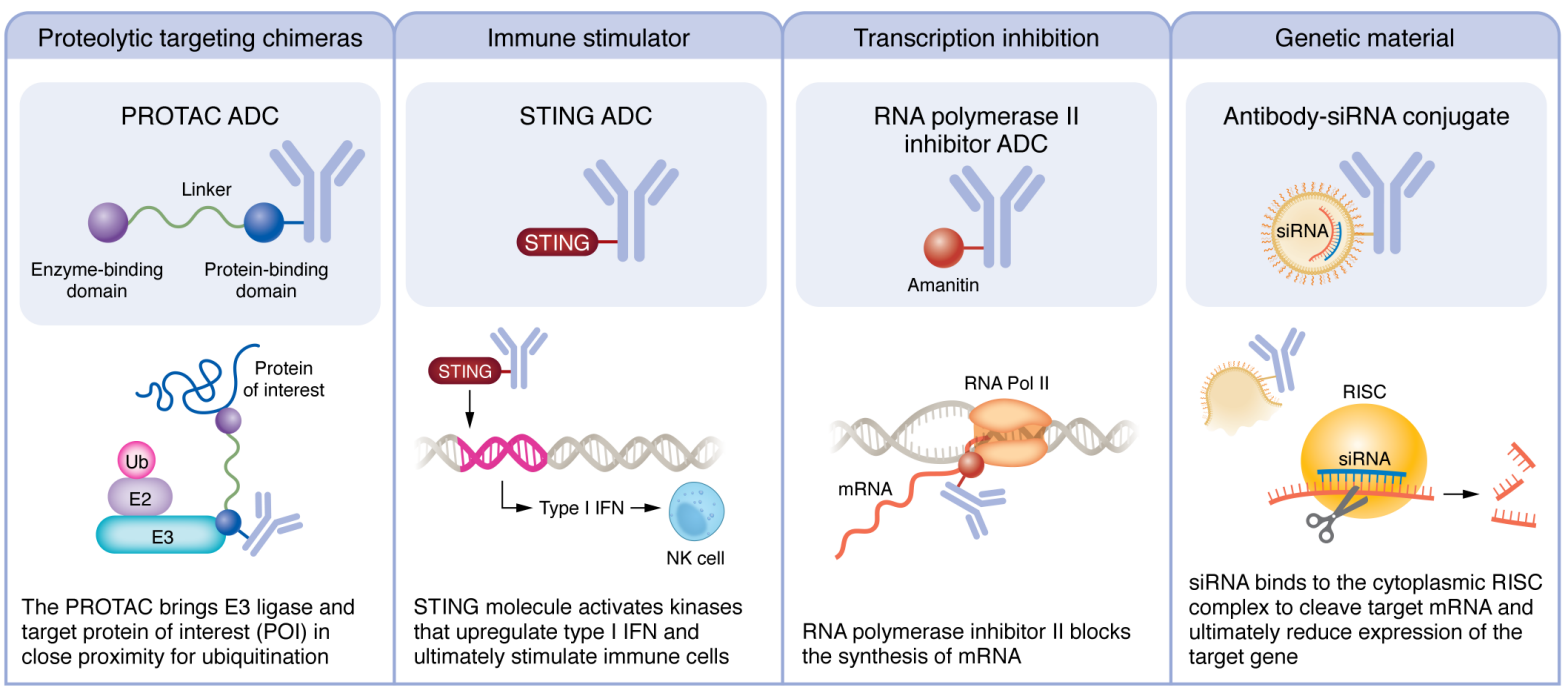
Payloads under investigation. The modular design of ADCs allows for payloads to be used interchangeably. Broad categories of payloads under investigation include proteolysis-targeting chimeras, immune stimulators, transcription inhibition, and genetic materials.

Modifications to the payload are another strategy to enhance antitumor effect and overcome resistance (Figure 3). A class of ADCs under the category of immune stimulator antibody conjugates (ISACs) are being developed to deliver targeted immune activators into tumors to increase effector immunity at the TME (86). NJH395 is an example of an ISAC composed of a TLR7 agonist conjugated to an HER2 antibody (87). A phase I clinical trial in patients with HER2-positive non-breast advanced malignancies has demonstrated intratumoral immune modulation with increased type II IFN-γ and subsequent CD8^+^ T cell infiltration in the TME; however, these findings did not result in a clinical response (87). Agonists of the cyclic GMP-AMP synthase (cGAS)/stimulator of interferon genes (STING) pathway are another payload target being evaluated preclinically to promote antitumor immunity through activation of T cells and NK cells (88, 89). The STING pathway is activated when cytosolic dsDNA binds to cGAS to produce cyclic GAMP (cGAMP). Upon stimulation by cGAMP, the STING molecule activates kinases that upregulate the expression of type I IFN, ultimately stimulating immune cells such as DCs, T cells, and NK cells (90). The biggest limitation of STING agonists is the risk of inducing excess cytokine release; thus, the potential of concentrating the STING payload in tumors via an ADC is especially promising. Wu et al. developed a STING agonist, IMSA172, which is conjugated to a tumor-targeting antibody against epidermal growth factor receptor (EGFR) (88). Administration of this STING ADC in mouse melanoma tumor models suppressed tumor growth. Furthermore, when the STING ADC was combined with anti–PD-L1 antibody, tumor growth was completely suppressed, suggesting synergy between these two treatments (88). In evaluating the mechanism through which STING ADCs promote antitumor immunity, tumors and draining lymph nodes of treated and untreated mice were collected and analyzed via fluorescence-activated cell sorting to identify immune cell populations and activation markers. The study found that the STING ADC activated both CD4^+^ and CD8^+^ T cells and increased the percentage of CD8^+^ T cells in the tumors (88). While the total number of NK cells was not changed by the STING ADC, the percentage of activated CD69^+^ NK cells showed a marked increase after therapy, which likely contributed to the antitumor effects. This finding also speaks to the heterogeneity of NK cells within the TME, which has implications in immunotherapy design and clinical practice (56, 91–94).

Proteolysis-targeting chimeras (PROTACs) are compounds composed of an E3 ligase ligand, a small chemical linker, and a ligand against a protein of interest (POI) with the ability to direct polyubiquitination and proteasome-mediated degradation of the POI. To date, PROTACs have been tested in 19 phase I or II clinical trials, with multiple others in investigational new drug (IND)–enabling studies (95). PROTACs maintain high therapeutic potential because they can distinctively target intracellular proteins that other small-molecular inhibitors are unable to reach.

In breast cancer, PROTACs have been used to target the receptors ER and HER2 (96). The PROTAC ARV-471, developed by Arvinas and Pfizer, is being evaluated in phase I/II trials in breast cancer as an oral ER degrader, and preclinical efforts to target HER2 have been effective in degrading HER2 to slow tumor growth (97). While promising, PROTACs are not tissue specific, and thus degradation of off-target proteins could be detrimental and thus dose-limiting. To optimize their therapeutic window, PROTACs could be conjugated to ADCs on or near the E3 ligase ligand of the PROTAC to improve specificity (98). As a proof-of-concept study, Maneiro et al. designed a trastuzumab-PROTAC conjugate to selectively target BRD4 for degradation in HER2-positive breast cancer cell lines. This study showed that the PROTAC-ADC specifically degraded BRD4 in HER2-positive cells, with no degradation observed in HER2-negative cells (99).

Another promising approach to ADC payload modification uses an RNA polymerase II inhibitor as the payload. RNA polymerase II inhibitors arrest the cellular transcription process and protein synthesis, leading to cell death. The benefit of these RNA polymerase inhibitors is that the cytotoxicity activity occurs regardless of the proliferation status of the cancer cell, unlike with microtubule- or DNA-targeting therapies (100). Thus, inhibiting RNA polymerase could be an attractive approach to target dormant cancer cells, which are in a G_0_ quiescent state and do not undergo active proliferation (101). HDP-101 is an RNA polymerase II ADC targeting anti–B cell maturation antigen (anti-BCMA) with an amanitin derivative and has shown ability to block tumor growth in both proliferating and resting multiple myeloma BCMA-positive cells (102). This early study provides promise for future work to target and eliminate dormant breast cancer cells, whose presence has strong correlation with later recurrence (103–105).

Antibody-oligonucleotide conjugates have been described to deliver DNA or RNA silencing nucleotides like siRNA (106). The Baumer laboratory has coupled siRNAs to an antibody against EGFR to deliver siRNA and silence KRAS expression (107). However, challenges remain with conjugation of the oligonucleotide/nanoparticle due to size and charge of the nucleic acids (106). Future work will involve improving the process of conjugation and delivery into cells.

Finally, dual-drug-conjugated ADCs are being constructed to evaluate the simultaneous delivery of two payloads to overcome tumor heterogeneity and ADC resistance (108). Investigational in vitro studies have found that simultaneous delivery of the conjugated payloads has greater efficacy than single-drug ADCs in resistant and heterogeneous HER2-expressing cell lines (108). It is hypothesized that the killing of cancer cells by the more potent payload helps the co-conjugated payload exert the bystander effect. Additionally, these dual-drug ADCs have greater antitumor effect than coadministration of single-drug ADCs. Dual-drug ADCs were found to accumulate in the tumor more effectively, likely because two single-drug ADCs would lead to binding competition and decreased internalization of the ADC (108). This payload class is likely to open a new avenue for ADCs with the benefit of further addressing tumor heterogeneity. At the time of this writing there is a rapidly expanding list of ADCs that are currently in the pipeline (Supplemental Table 1; supplemental material available online with this article; https://doi.org/10.1172/JCI172156DS1).

## Conclusions and future directions

A better mechanistic understanding of how the TME responds to ADC treatment could lead to durable responses because of amplification of the immune system. The next era of ADCs will be focused on strategies to overcome resistance and enhance efficacy by pairing ADCs with signaling pathway inhibitors, ICIs, other TME modifiers, and emerging payloads. However, delivery of either monotherapies or combination therapies still lacks an effective means of patient selection. This challenge is particularly critical in the present era where certain ADCs, such as T-DXd and SG, surpass limitations of our traditional breast cancer subtypes. Thus, the development of biomarkers, such as CTCs, to determine the patient population likely to benefit from ADC treatment becomes imperative. To uncover such biomarkers, it is important to conduct studies using advanced analytic techniques of the entire microenvironment, such as single-cell and spatial “omics” and functional assays with tumor organoids and immune cells (109–114). Understanding how signaling networks change across cell types and how ADCs augment the function of effector immune cells or decrease regulatory immune cells could lead to new biomarker development and therapies.

## Supplementary Material

Supplemental table 1

## Figures and Tables

**Table 2:**
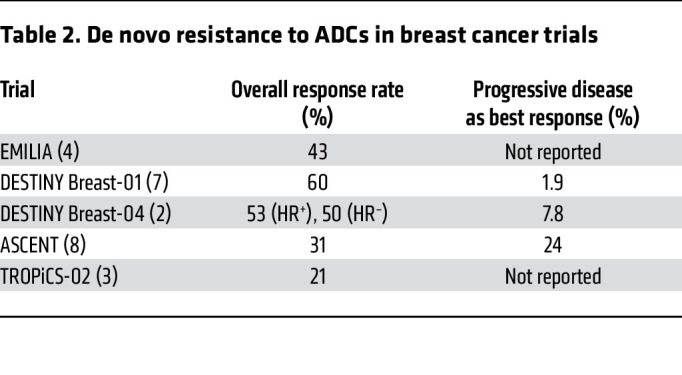
De novo resistance to ADCs in breast cancer trials

**Table 1:**
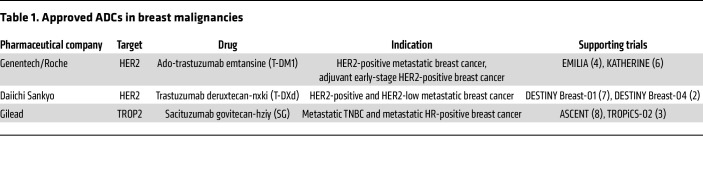
Approved ADCs in breast malignancies

## Version 1 09/15/2023

Electronic publication
