# Supplementary material for: Antibody-drug conjugates in breast cancer: overcoming resistance and boosting immune response
Source: J Clin Invest. 2023 Sep 15;133(18):e172156. doi: 10.1172/JCI172156 (PMC10503805; doi:10.1172/JCI172156)
Supplement: Supplemental table 1 [file jci-133-172156-s154.pdf]

Pharmaceutical Company	Target	Drug	Combination	Indication	Phase	Identifier	Results
Seagen	CD30	Brentuximab vedotin	+ Pembrolizumab	R/R Metastatic Tumors with PD-L1 positivity	I/II	NCT04609566	None, Primary Endpoint: ORR
Seagen	Nectin-4	Enfortumab vedotin monotherapy		Advanced/Metastatic Solid Tumors	I/II	NCT04225117	None, Primary Endpoint: ORR
Seagen	LIV-1	Ladiratuzumab vedotin	+/- Pembrolizumab	Metastatic TNBC and R/R Metastatic breast cancer	I/II	NCT03310957	None, Primary Endpoint: ORR
Seagen	CD228A	SGN-CD228A		R/R Solid Tumors	I	NCT04042480	None, Primary Endpoint: number of AE, lab abnormalities, DLTs
Seagen	Integrin beta-6	SGN-B6A		R/R Solid Tumors	I	NCT04389632	None, Primary Endpoint: AE, lab abnormalities, DLTs
Seagen	Sialyl Thomsen-nouveau (STn)	SGN-STNV		R/R Solid Tumors	I	NCT04665921	None, Primary Endpoint: AE, lab abnormalities, DLTs
Seagen	B7-H4	SGN-B7H4V		R/R Solid Tumors	I	NCT05194072	None, Primary Endpoint: AE, lab abnormalities, DLTs
Seagen	PD-L1	SGN-PDL1V		Advanced Solid Tumors	I	NCT05208762	None, Primary Endpoint: AE, lab abnormalities, DLTs
Seagen/Genetech	HER2	Trastuzumab Emtansine (T-DM1)	+ Tucatinib	Metastatic HER2+	II	NCT05673928	None, Primary Endpoint: intracranial antitumor activity
Daiichi Sankyo	HER2	Trastuzumab deruxtecan (T-DXd)	+ AZD5305 (PARPi)	Solid Tumors	I	NCT04644068	None, Primary Endpoint: AE, DLTs
Daiichi Sankyo	TROP2	Dato-DXd	+ AZD5305 (PARPi)	Solid Tumors	I	NCT04644068	None, Primary Endpoint: AE, DLTs
Daiichi Sankyo	HER3	Patritumab deruxtecan (HER3-DXd)		HER3+ Metastatic Breast Cancer	I	NCT04965766	None, Primary Endpoint: ORR
			+ Durvalumab				Primary Endpoint: AE
			+ Paclitaxel				R2PD combinations were the standard doses of each individual drug
			+ Pertuzumab				
Daiichi Sankyo	HER2	Trastuzumab deruxtecan (T-DXd)	+ Tucatinib	HER2+ Metastatic Breast Cancer	I/II	NCT04538742	
Daiichi Sankyo	HER2	Trastuzumab deruxtecan (T-DXd) vs. Trastuzumab Emtansine (T-DM1)		Early Stage HER2+ Breast Cancer with Residual Disease	III	NCT04622319	None, Primary Endpoint: IDFS
			+ Trastuzumab				Primary Endpoint: AE
			+ Durvalumab				R2PD combinations were the standard doses of each individual drug
			+ Paclitaxel				
			+ Capivasertib				
			+ Anastrazole				
			+ Fulvestrant				
Daiichi Sankyo	HER2	Trastuzumab deruxtecan (T-DXd)	+ Capecitabine	Metastatic HER2 low Breast Cancer	I	NCT04556773	
Daiichi Sankyo	TROP2	Datopotamab deruxtecan (Dato-DXd)		Metastatic TNBC Not Candidate for Immunotherapy	III	NCT05374512	None, Primary Endpoint: PFS, OS
Daiichi Sankyo	TROP2	Datopotamab deruxtecan (Dato-DXd)		Metastatic HR+, HER2 Negative Breast Cancer	III	NCT05104866	None, Primary Endpoint: PFS, OS
			+ Durvalumab				
			+ Capecitabine				
Daiichi Sankyo	TROP2	Datopotamab deruxtecan (Dato-DXd)	+ Pembrolizumab	Early Stage TNBC with Residual Disease	III	NCT05629585	None, Primary Endpoint: IDFS
ADC Therapeutics	AXL	Mipasetamab uzoptirine (ADCT-601)		Advanced Solid Tumors	I	NCT05389462	None, Primary Endpoint: AE, DLTs, dose reduction, dose interruption
ADC Therapeutics	KAAG1	ADCT-901		Advanced Solid Tumors	I	NCT04972981	None, Primary Endpoint: AE, DLTs, dose reduction, dose interruption
Sanfoni	CEACAM5	Tusamitamab ravtansine	+/- Gemcitabine	Metastatic Breast Cancer	II	NCT04659603	None, Primary Endpoint: antitumor activity, recommended dose
Gilead	TROP2	Sacituzumab Govitecan		Metastatic HR+, HER2 negative Breast Cancer	III	NCT03901339	Primary Endpoint: PFS
							SG (vs TPC) improved median PFS (5.5 vs 4.0m)
Gilead	TROP2	Sacituzumab Govitecan		Untreated Metastatic TNBC, PDL-1 negative	III	NCT05382299	None, Primary Endpoint: PFS
Gilead	TROP2	Sacituzumab Govitecan		Untreated Metastatic TNBC, PDL-1 positive	III	NCT05382286	None, Primary Endpoint: PFS
Gilead	TROP2	Sacituzumab Govitecan	+ Alpelisib	Metastatic HER2 negative Breast Cancer	I	NCT05143229	None, Primary Endpoint: R2PD
Gilead	TROP2	Sacituzumab Govitecan	+ Pembrolizumab	Metastatic TNBC (PDL-1 positive)	III	NCT04230109	None, Primary Endpoint: pCR
Gilead	TROP2	Sacituzumab Govitecan	+ Pembrolizumab	Metastatic TNBC (PDL-1 negative)	II	NCT04468061	None, Primary Endpoint: PFS
Gilead	TROP2	Sacituzumab Govitecan	+/- Pembrolizumab	Neoadjuvant Early Stage TNBC	II	NCT04230109	Primary Endpoint: pCR
							pCR rate with SG alone was 30%
Gilead	TROP2	Sacituzumab Govitecan	+ Pembrolizumab	Early Stage Immunotherapy Resistant TNBC	II	NCT05675579	None, Primary Endpoint: AE
Gilead	TROP2	Sacituzumab Govitecan	+ Pembrolizumab	Early Stage TNBC with Residual Disease	III	NCT05633654	None, Primary Endpoint: IDFS
Gilead	TROP2	Sacituzumab Govitecan	+ Pembrolizumab	Metastatic HR+, HER2 negative Breast Cancer	II	NCT04448886	None, Primary Endpoint: PFS
Gilead	TROP2	Sacituzumab Govitecan	+ Magrolimab	Metastatic TNBC	II	NCT04958785	None, Primary Endpoint: AE, DLT, PFS, ORR

Gilead	TROP2	Sacituzumab Govitecan		HER2 negative Breast Cancer with Brain Metastases	II	NCT04647916	None, Primary Endpoint: ORR
Gilead	TROP2	Sacituzumab Govitecan		Early Stage TNBC with Residual Disease	III	NCT04595565	Primary Endpoint: IDFS<br>SG showed a higher rate of AEs compared to TPC
G1 Therapeutics	TROP2	Sacituzumab Govitecan	+ Trilaciclib	Metastatic TNBC	II	NCT05113966	None, Primary Endpoint: PFS
Pfizer	TROP2	Sacituzumab Govitecan	+ Talazoparib	Metastatic TNBC	I/II	NCT04039230	Primary Endpoint: DLT
							Staggered dosing of SG and PARPi was feasible with evidence of clinical activity with objective responses
Pfizer	TROP2	Sacituzumab Govitecan	+ Avelumab	Metastatic TNBC	II	NCT03971409	None, Primary Endpoint: BORR
Pfizer	HER2	Trastuzumab Emtansine (T-DM1)	+ Palbociclib	Metastatic HER2 positive Breast Cancer	II	NCT03530696	None, Primary Endpoint: PFS
Genentech	HER2	Trastuzumab Emtansine (T-DM1)		Stage I HER2 positive Breast Cancer	II	NCT04893109	None, Primary Endpoint: CRT, DFS
Genentech	TROP2	Sacituzumab Govitecan	+ Atezolizumab	Early Stage TNBC with Residual Disease	II	NCT04434040	None, Primary Endpoint: rate of cfDNA
Genentech	HER2	Trastuzumab Emtansine (T-DM1)	+ Vinorelbine	Metastatic HER2 positive Breast Cancer	I/II	NCT02658084	None, Primary Endpoint: MTD, PFS, AE
Hoffmann-La Roche	HER2	Trastuzumab Emtansine (T-DM1)	+ Atezolizumab	Metastatic HER2 positive, PDL-1 positive metastatic breast cancer	III	NCT04740918	None, Primary Endpoint: PFS, OS
Hoffmann-La Roche	HER2	Trastuzumab Emtansine (T-DM1)	+ Atezolizumab	HER2 positive Breast Cancer with Residual Disease	III	NCT04873362	None, Primary Endpoint: IDFS
Hoffmann-La Roche	HER2	Trastuzumab Emtansine (T-DM1)	+ Atezolizumab	Metastatic HER2 positive Breast Cancer	II	NCT02924883	Primary Endpoint: PFS, AE
							Addition of atezolizumab did not statistically improve PFS (8.2m vs 6.8m)
Merck Sharp & Dohme	HER2	Trastuzumab Emtansine (T-DM1)	+ Pembrolizumab	Metastatic HER2 Positive Breast Cancer	I	NCT03032107	Primary Endpoint: AE
							No DLT, thus R2PD is full dose pembrolizumab + T-DM1
							ORR was 20% and median PFS was 9.6 months
Novartis	HER2	Trastuzumab Emtansine (T-DM1)		Metastatic HER2 Positive Breast Cancer	I	NCT02038010	None, Primary Endpoint: DLTs, MTD
Immunogen	ADAM9	IMGC936		Solid Tumors	I	NCT04622774	None, Primary Endpoint: DLT, AE, ORR

Supplemental Table 1. **ADC Pipeline in Breast and Solid Malignancies**

Abbreviations

DLTs: dose limiting toxicities
AE: number of adverse events
MTD: maximum tolerated dose
CRT: clinically relevant toxicity
ORR: overall response rate
BORR: best overall response rate
PFS: progression free survival
OS: overall survival
IDFS: invasive disease free survival
R2PD: recommended phase 2 dose
cfDNA: cell free Deoxyribonucleic Acid
TPC: treatment of physician's choice
